# Effects of Long-Term Meditation Practices on Sensorimotor Rhythm-Based Brain-Computer Interface Learning

**DOI:** 10.3389/fnins.2020.584971

**Published:** 2021-01-21

**Authors:** Xiyuan Jiang, Emily Lopez, James R. Stieger, Carol M. Greco, Bin He

**Affiliations:** ^1^Department of Biomedical Engineering, Carnegie Mellon University, Pittsburgh, PA, United States; ^2^Department of Biomedical Engineering, University of Minnesota, Minneapolis, MN, United States; ^3^Department of Psychiatry, University of Pittsburgh, Pittsburgh, PA, United States

**Keywords:** EEG, electroencephalogram, mindfulness, meditation, BCI, brain-computer interface

## Abstract

Sensorimotor rhythm (SMR)-based brain–computer interfaces (BCIs) provide an alternative pathway for users to perform motor control using motor imagery. Despite the non-invasiveness, ease of use, and low cost, this kind of BCI has limitations due to long training times and BCI inefficiency—that is, the SMR BCI control paradigm may not work well on a subpopulation of users. Meditation is a mental training method to improve mindfulness and awareness and is reported to have positive effects on one’s mental state. Here, we investigated the behavioral and electrophysiological differences between experienced meditators and meditation naïve subjects in one-dimensional (1D) and two-dimensional (2D) cursor control tasks. We found numerical evidence that meditators outperformed control subjects in both tasks (1D and 2D), and there were fewer BCI inefficient subjects in the meditator group. Finally, we also explored the neurophysiological difference between the two groups and showed that the meditators had a higher resting SMR predictor, more stable resting mu rhythm, and a larger control signal contrast than controls during the task.

## Introduction

Decades of research have sought to find alternative methods of communication between the human brain and the outside world. With the ever-growing knowledge in the neuroscience field, scientists have designed the brain–computer interface (BCI) to achieve this goal (Wolpaw et al., 2002; He et al., 2020). A BCI attempts to recognize the user’s intent by decoding her/his neurophysiological signals and then converts this intent into commands to control objects, such as a cursor on a computer screen (Wolpaw et al., 1991; Trejo et al., 2006), a quadcopter (LaFleur et al., 2013), or a robotic arm in space (Meng et al., 2016; Edelman et al., 2019).

One of the main goals for the BCI is to help people suffering from various kinds of neuromuscular diseases, such as amyotrophic lateral sclerosis, stroke, and spinal cord injury (Armour et al., 2016) to regain a certain degree of movement ability (Rebsamen et al., 2010; Ang et al., 2015). Despite the limited ability to move, cognitive ability in this population remains partially or fully intact. Therefore, it would be a significant improvement in the quality of life if these individuals could use a BCI to complete daily life tasks.

There are many types of BCI based on various recording techniques and signals extracted. In this work, we focus on the sensorimotor rhythm (SMR)-based BCI, which uses electroencephalogram (EEG) to detect scalp electrical signals and decode motor intention (Yuan and He, 2014; He et al., 2015). The EEG-based SMR BCI has multiple merits, such as non-invasiveness, ease of use, relatively low cost, and high temporal resolution (He et al., 2020). This is particularly true when only a few electrodes are used (Clerc et al., 2016). The SMR or mu rhythm in EEG is generated by the synchronized electrical brain activity over the motor cortex area and has a frequency range of around 8–12 Hz (Pfurtscheller et al., 2006; Bernier et al., 2007). In BCI applications, the frequency band centered at 12 Hz (Royer et al., 2010; Doud et al., 2011; Cassady et al., 2014; Meng et al., 2016, 2018; Stieger et al., 2020) was shown to be effective in SMR control. Event-related desynchronization (ERD) occurs when the amplitude of mu rhythm decreases in response to a person moving or imagining moving her/his body (Pfurtscheller and Aranibar, 1979). On the other hand, when a person stops moving or imagining moving, the amplitude of mu rhythm increases, which is called event-related synchronization (ERS). SMR based BCI is a well-established BCI modality, and it has been demonstrated that people can perform multidimensional cursor control (McFarland et al., 2010; Meng et al., 2018), drone control (Royer et al., 2010; Doud et al., 2011; LaFleur et al., 2013), wheelchair control (Galán et al., 2008; Huang et al., 2012), and robotic arm control (Meng et al., 2016; Edelman et al., 2019) with SMR BCI.

Despite the progress of SMR-based BCI, challenges exist. Two primary limitations of SMR-based BCI are the system’s need for long training times and BCI inefficiency, where the SMR BCI control paradigm may not work on around 20% of the system’s users (Blankertz et al., 2010). The latter could be further developed as a subject variability issue: there exists a large variability of SMR BCI performance among the population. Efforts have been made to investigate its cause and solution (Ahn and Jun, 2015; Jeunet et al., 2016). For instance, Guillot et al. (2008) found that good MI performers have an increased ability to recruit MI-related brain network, and Sannelli et al. (2008) found that BCI inefficient subjects usually have higher intrinsic noise, i.e., the noise in the data which can overshadow the class-related information; Ahn and Jun (2015) did a systematic review of literature on SMR BCI inefficiency and found that these subjects typically have less developed brain networks for motor skills; Jeunet et al. (2016) further summarized the factors influencing SMR BCI as a relationship to the technology, attention, and spatial abilities.

Attention has been focused on developing better decoding algorithms and recording techniques (Lotte and Guan, 2011) for SMR BCI, i.e., from the “computer” perspective of BCI. However, less attention has been drawn to enhancing people’s ability to generate more decodable EEG signals, i.e., from the “brain” side. For the latter, the high-level goal is to determine, given the same BCI system, if there exists a subpopulation that is better able to control it and if a certain kind of training or intervention could be developed to equip neurotypical people with this BCI control ability.

In the search for optimal mental training methods to potentially improve SMR BCI control, meditation is of interest due to its ability to alter brain plasticity and influence spatial–temporal brain activity, which in turn, are important components of SMR BCI control (Chan and Woollacott, 2007; Tang et al., 2007; Moore and Malinowski, 2009; Debarnot et al., 2014). As summarized in Debarnot et al. (2014), one of the most important effects of meditation is enhanced attention control, such as orienting attention (van den Hurk et al., 2010) and conflict monitoring (Jha et al., 2007). In terms of the influence of meditation on brain rhythms, Halsband et al. (2009) found that in hypnotic and mindfulness meditation states, there exist a modulation of alpha, gamma, and theta band brain rhythms, including but not limited to the sensorimotor area, which indicates the ability of meditation to alter motor-related spatial–temporal brain activity. In Kerr et al. (2011a), mindfulness training was found to enhance MEG alpha power modulation in the primary somatosensory cortex (SI).

With growing evidence suggesting that meditation brings enhanced attention and brain rhythm control, it is reasonable to hypothesize that people with meditation experience would develop a better ability to control SMR-based BCI. Indeed, previous work has investigated the effect of meditation on SMR BCI cursor control (Cassady et al., 2014; Tan et al., 2014, 2015; Kober et al., 2017; Stieger et al., 2020) or just generating ERD/ERS without controlling a BCI system (Kerr et al., 2011a,b, 2013; Rimbert et al., 2019). Similar to what Tang et al. (2015) summarized for the neuroscience aspect of meditation studies, efforts to study the meditation effect on SMR BCI could be divided into two categories, longitudinal studies and cross-sectional studies:

1. Previous longitudinal studies separated meditation-naïve subjects into a meditation group and a control group, with the meditation group receiving meditation training and the control group receive either active control tasks or no specific task (Mahmoudi and Erfanian, 2006; Tan et al., 2014, 2015; Botrel and Kübler, 2019; Stieger et al., 2020). After that, BCI performance and/or neurophysiological difference between the two groups was assessed. For example, a series of studies by Tan et al. (2014, 2015) and Ramli et al. (2019) described the effect of 4 weeks mindfulness meditation on SMR BCI performance, that mindfulness meditation improved BCI performance and was correlated with activation in the frontal–parietal region in functional magnetic resonance imaging during motor imagery. In Mahmoudi and Erfanian (2006), the mental practice of MI and concentration procedures improved the offline classification of MI in multiple EEG electrodes, such as C3 in the primary motor cortex area and F3 in the frontal area. In Kerr et al. (2013), they discovered that an 8 weeks mindfulness-based stress reduction training served to optimize attentional modulation of 7–14 Hz alpha rhythm in the primary sensory neocortex. Rimbert et al. (2019) found that the hypnotic state changes the sensorimotor beta rhythm during the ERD period, whereas the ERS in the mu and beta band remains unchanged. In Stieger et al. (2020), an 8 weeks MBSR class improved SMR BCI accuracy via modulation of the volitional resting-state high alpha EEG rhythm.
2. In contrast, cross-sectional studies have investigated the difference in BCI/neurofeedback learning between people who already have meditation experience and meditation naïve subjects (Cassady et al., 2014; Kober et al., 2017). In Cassady et al. (2014), the meditation group was shown to have better results compared with the control group in terms of performance, learning speed, and information transfer rate. However, most of the claims in this study focused on the behavior difference. A more in-depth analysis of the neurophysiological difference is needed. Another question left unanswered is whether meditators are also better at more complex tasks, such as two-dimensional (2D) cursor control. A typical 2D cursor control paradigm is achieved by having the subject use left/right (LR) motor imagery to control LR cursor movement, bilateral hand motor imagery to go up, and rest to go down. Successful 2D cursor control requires the subject to carefully balance the strength of LR motor imagery and, therefore, is more challenging than 1D control. Because meditators are trained to control their attention, it is of interest to see if the BCI learning difference between meditators and non-meditators in a 2D BCI task would be even larger compared with 1D tasks and if there is any difference between the LR and up/down (UD) within the 2D compared with the 1D version of LR and UD tasks. In another study, Kober et al. (2017) found that people who pray frequently had a higher ability to control the SMR, but the recording was limited to Cz electrode only, and the control dimension was limited to 1D.

Despite the abundance of literature reporting positive effects of meditation on SMR BCI control, there are also studies whose results only partially support (Stieger et al., 2020) or do not support such a hypothesis (Botrel and Kübler, 2019). For example, Stieger et al. (2020) found that after an 8 weeks mindfulness-based stress reduction training, subjects indeed had significant performance improvements in the UD task (both hands motor imagery to go up and rest to go down), but for the LR control task (LR-hand motor imagery) the effect was not significant. Botrel and Kübler (2019) found that week-long visuomotor coordination and relaxation training did not improve SMR-based BCI performance. One of the reasons for this kind of disagreement may be a dose-effect, meaning that it might take a longer meditation time to affect BCI learning in a significant manner.

With these questions in mind, we recruited experienced meditators and controls and investigated the difference in SMR BCI learning between these two groups in both 1D and 2D tasks. The aims for this cross-sectional study are as follows: First, to verify the conclusions in the pilot study (Cassady et al., 2014) that meditators had better learning in SMR BCI with an independent investigation; second, to explore the behavior difference between the two groups in a more complex 2D task; and third, to investigate the neurophysiological difference between these two groups.

## Materials and Methods

### Participants

The experimental procedures involving human subjects described in the current study were approved by the Institutional Review Board (IRB) of Carnegie Mellon University with study ID STUDY2017_00000430, and all participants provided written informed consent. Subjects were recruited via flyers in the surrounding area and an email sent out to local mindfulness groups. We utilized a single-blind two-group experimental design, with a meditation group and a control group. The experimenters did not know the identity of the subject in relation to their meditation experience throughout the whole experiment. We achieved this blinding through the following: (1) we asked two other researchers at our lab to refer potential subjects (both meditators and controls) to an unblinded research assistant (screener) and not to the blinded experimenters; (2) these potential subjects were screened for inclusion/exclusion by this screener whose only involvement in the study was to conduct screening; (3) during the consent process and survey, the experimenter asked the subject to cover any information related to meditation experience when submitting the paperwork; thus, the experimenter only knew information unrelated to meditation (age, sex, name, etc.) about this subject after collecting these documents. (4) During the experiment, the experimenter remained unaware of the subjects’ meditation status and avoided any conversation related to meditation with the subject throughout the entire six sessions.

The meditation group consisted of 16 healthy subjects (age = 38.5 ± 15.7 years) with a history of meditation practice, as evaluated by a questionnaire regarding personal meditation practice completed before experimentation. To be accepted into the meditator group, individuals had to cite at least a year of frequent and consistent practice, with most subjects having 2 or more years of consistent practice. Most of the meditators’ practices belong to the subgroup of Vipassana, Zen, Mindfulness, and Buddhism. The control group consists of 19 healthy individuals (age = 25.6 ± 9.4 years) with no prior meditation experience. Both groups had no prior BCI experience. We continually asked participants to describe their motor imagery strategies. If these strategies diverged from the kinesthetic motor imagery they were asked to perform, we reminded them to focus on the sensations and intention behind the imagined motion of their hands. We excluded one subject (identity: meditator) from the analysis because she/he expressed resistance to performing the required motor imagery and was not able to provide a concrete strategy when asked. Subjects’ demographic information is summarized in Supplementary Table 1.

### Surveys to Measure Mindfulness

In the first session, we asked subjects to fill out two surveys before the BCI experiment. Both surveys aim to measure one’s level of mindfulness. The first survey is called the Freiburg Mindfulness Inventory (FMI) (Walach et al., 2006), which has 14 statements, such as “I am open to the experience of the present moment.” The subject was asked to use a 1–4 scale to indicate how often she/he has such experience. The FMI score was calculated by summing up the answers to each question with a proper recode of one question (Walach et al., 2006). The second survey is called Day-to-Day Experiences (Brown and Ryan, 2003), which has 15 questions, such as “I find it difficult to stay focused on what’s happening in the present;” the subject was asked to use a 1–6 scale to indicate how often she/he has such experience. The Mindful Attention Awareness Scale (MAAS) was calculated by averaging answers to each question in this Day-to-Day Experiences survey. In both surveys, a higher score indicates a higher level of mindfulness.

### Data Acquisition and Brain–Computer Interface Cursor Control Task

Subjects in both groups went through six sessions of BCI training within 4–6 weeks, with at least 1 session per week. Each experimental session lasted about 2 h, with a 9 min break in the middle. EEG data were recorded throughout the session using the Neuroscan SynAmps system with 64-channel EEG QuikCap (Neuroscan Inc., Charlotte, NC). The sampling frequency was set to 1,000 Hz, and the impedance was kept below 5 kΩ during the preparation. The experimenter checked the impedance in the break to make sure it remained below 5 kΩ. In addition, to minimize the influence of artifact on the EEG data, we monitored the behavior of the subject and the recorded waveform. We restarted this run if we found the subject moved a lot or the real-time EEG signal became noisy.

**FIGURE 1:**
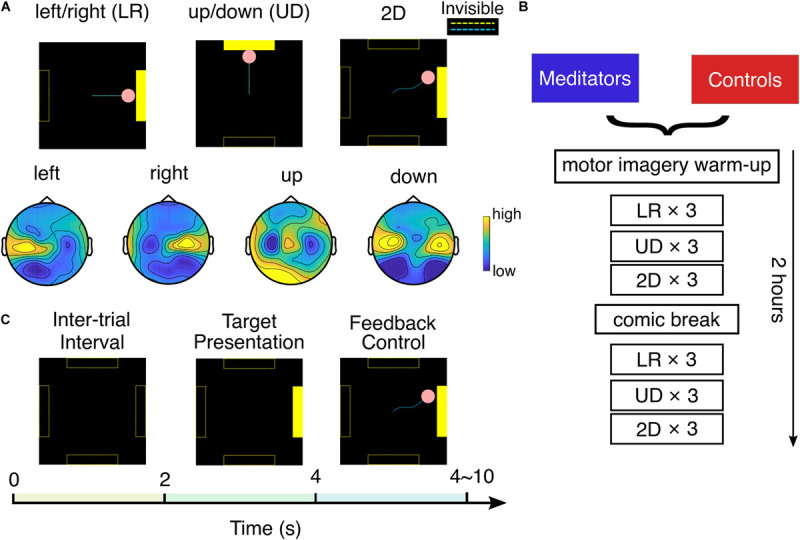
Experimental setup. **(A)** Top: three experiment tasks and typical cursor trajectories in left/right (LR) control, up/down (UD) control, and 2D control. Dashed lines were invisible to the subject. Bottom: example topology of mu rhythm band power in each motor imagery class. **(B)** Experiment flow of one session. **(C)** Each trial consists of 2 s of intertrial interval, 2 s of target presentation, and 0–6 s of BCI feedback control.

The experiment setup is shown in Figure 1. Each session began with a 5 min warmup task, where the subject was instructed to perform left- or right-hand motor imagery by focusing on imagining the sensations and intention of opening/closing the LR hand.

After that, the subject was asked to perform BCI cursor control of three different tasks: LR, UD, and 2D, by moving the cursor to the corresponding bar with motor imagery. This experiment flow is detailed in Figures 1A,B. In the LR task, subjects were told to imagine opening/closing the LR hand as they practiced in the warm-up to move the cursor to the LR, to hit a bar that appears randomly at the right or left side of the screen. The vertical position of the cursor was fixed in the middle of the screen in the LR task. After subjects performed three runs of LR BCI, with each run consisting of 25 trials, a similar explanation was given for the UD BCI task, except they were instructed to imagine both hands simultaneously opening and closing to move the cursor up, and to rest, in other words, try to clear their minds to move the cursor down. The cursor’s horizontal position was fixed at the middle of the screen in the UD task. After subjects performed three runs of UD BCI, they moved onto the 2D task, in which the same instructions were implemented to move the cursor up, down, left, or right according to which bar appeared on the screen. In the 2D task, the cursor is free to move in any direction within the screen boundary. After one block (three runs) each of LR, UD, and 2D BCI, the subjects were given a 9 min break in which they were instructed to read and rate comics by pressing a key on the keyboard. This standard “break task” ensures that subjects use the same approach to relax. After the break, they completed one more block each of LR, UD, and 2D BCI. In total, at each session, a subject completes six runs (25 trials each run, takes ∼3 min each run) of LR, UD, and 2D tasks.

We used the standard cursor task in BCI2000 (Schalk et al., 2004) to conduct the SMR BCI experiment mentioned earlier. The technical details of the classifier are presented as follows: The spectral amplitude of the small Laplacian filtered C3 and C4 electrodes were estimated using autoregressive (AR) methods in a 3 Hz bin (Stieger et al., 2020) surrounding 12 Hz (Meng et al., 2016, 2018). After that, for the horizontal motion, a control signal was calculated by taking the AR amplitude difference between two electrodes (C4 – C3), and for the vertical motion, it was calculated by summing up the AR amplitude of two electrodes (C4 + C3). This control signal was further subtracted by an offset and multiplied with a gain value to make the normalized control signal zero mean and unit variance. The pink cursor acted as the feedback to the subject; the normalized control signal determined its speed, and its position was updated every 40 ms. The gain and offset values were reset when performing a new task (LR, UD, and 2D) and after the break. As shown in Figure 1C, each trial starts with a 2 s intertrial interval where the screen was black; then, the yellow target bar appears randomly at one of the possible locations for 2 s; after that, the subject was able to use MI to control the cursor (bar still visible). The length of the feedback control varied between 0 and 6 s and depended on if and when the cursor hits the bar. There could be three possible outcomes for each trial: the cursor hits the correct bar (hits, the cursor turns yellow), the cursor hits the incorrect bar (misses, the cursor does not change color), or the cursor does not contact any target within 6 s (timeout, the cursor does not change color).

### Performance Metric

We quantify the performance using percent valid correct (PVC) (Cassady et al., 2014; Meng et al., 2016; Edelman et al., 2019), which is the ratio between the number of hit trials and number of hit trials plus the number of missed trials. To reduce the influence of large subject variability in SMR BCI, in the analysis, we excluded outlier subjects: we compute the averaged percent valid correct (PVC) across the six sessions for a subject as the performance, for LR, UD, and 2D, respectively. After that, for each task (LR, UD, and 2D), we identified any subjects that are ± 2.5 median absolute deviations from the median of the whole sample (Leys et al., 2013). Finally, we took the union of subjects identified in the three tasks as the excluded subjects. With this criterion, we identified one meditator and four controls as outliers. Together with the subject excluded due to not following the MI guideline (one meditator), the number of subjects involved in the analysis is 14 meditators and 15 controls. The information regarding the median performance and outlier subjects’ performance is shown in Supplementary Table 2.

### Offline Electroencephalogram Data Analysis

We bandpass filtered the EEG data using a Hamming window as a finite impulse response filter with the passband set between 1 and 100 Hz, then downsampled to 250 Hz. We identified and rejected noisy channels with high impedance by visual inspection; then, these channels were spherically interpolated. The EEG data were re-referenced to a common average. We attempted to remove potential eye blinking artifacts using independent component analysis and a template matching procedure. In addition, we also visually inspected trials with high data variance and excluded these trials in the analysis, as these trials have a higher probability of containing muscle artifact. After that, complex Morlet wavelet convolution was used to extract the power of the mu frequency band (3 Hz bin centered at 12 Hz).

The neurophysiological predictor or SMR predictor measures the difference between mu band power and the 1/*f* noise floor in a power-frequency plot for C3 and C4 (Blankertz et al., 2010). Concretely, the EEG power spectrum at rest could be fitted with the sum of a 1/*f* noise floor, *n*(*f*;λ,*k*_*n*_) and two Gaussian distributions, centered at mu rhythm and beta rhythm, *g*_α_(*f*;μ_α_,σ_α_) and *g*_β_(*f*;μ_β_,σ_β_). In this study, the power spectral density is equal to the mean of C3 and C4 band power after small Laplacian spatial filtering during the intertrial resting state, combining LR conditions and UD conditions.

$$\hat{P\,S\,D}\,\left(f;\lambda ,\sigma ,k\right)=n\,\left(f;\lambda ,k_{n}\right)+g_{\alpha }\,\left(f;\mu_{\alpha },\sigma_{\alpha }\right)+g_{\beta }\,\left(f;\mu_{\beta },\sigma_{\beta }\right)$$

$$n\,\left(f;\lambda ,k_{n}\right)=k_{n\,1}+\frac{k_{n\,2}}{f^{\lambda }}$$

$$g_{\alpha }\,\left(f;\mu_{\alpha },\sigma_{\alpha }\right)=k_{\alpha }\,N\,\left(f;\mu_{\alpha },\sigma_{\alpha }\right)$$

$$g_{\beta }\,\left(f;\mu_{\beta },\sigma_{\beta }\right)=k_{\beta }\,N\,\left(f;\mu_{\beta },\sigma_{\beta }\right)$$

The SMR predictor (dB) is calculated individually for C3 and C4 electrode mu rhythm band power after small Laplacian spatial filtering.

$$P\,r\,e\,d\,i\,c\,t\,o\,r=10\cdot l\,o\,g_{10}\,\frac{P\,S\,D\,\left(m\,u\right)}{n\,\left(m\,u\right)}$$

In the case where the algorithm could not find a curve to fit, we manually selected 5–10 representative data points to describe the 1/*f* noise floor function by following the trend of the PSD curve and fitted these points using *n*(*f*;λ,*k*_*n*_). We discard a subject and session pair if the PSD does not follow a 1/*f* decrease trend. The percentage of data points discarded was 10.5%.

We designed a method to calculate the control signal during task execution to be as close to the real condition as possible. Concretely, we first calculated the C3 and C4 electrode frequency band power after small Laplacian spatial filtering, denoted *P*_*C3*_ and *P*_*C4*_. Then, the raw control signal was calculated using the following equation:

$$C\,S_{r\,a\,w,L\,R}=\,P_{C\,4}-P_{C\,3}$$

$$C\,S_{r\,a\,w,U\,D}=\,P_{C\,4}+P_{C\,3}$$

Then, we applied a similar z-scored procedure to the raw control signal as the BCI 2000 platform,

$$C\,S_{r\,e\,a\,l}=G\times \left(C\,S_{r\,a\,w}-o\,f\,f\,s\,e\,t\right)$$

where *G* and *offset* are set to make the *CS*_*real*_ zero mean and unit variance. The difference between this offline z-score and the online approach is that the latter is causal and adaptive, i.e., *G* and *offset* is calculated via past 30 s of a window and change as time goes on. As shown in Supplementary Figure 1, we found that the control signal under this definition could better explain the variability of performance than the ERD/ERS method, i.e., band power during task execution divided by resting-state band power.

We quantify the contrast between two contexts in a task (e.g., left trials and right trials in LR task) using the Fisher score (Perdikis et al., 2018).

$$F\,S=\frac{\left|\mu_{1}-\mu_{2}\right|}{\sqrt{{\text{s}}_{1}^{2}+{\text{s}}_{2}^{2}}}$$

where μ_*1*_and μ_*2*_ are the means and ${\text{s}}_{1}^{2}$ and ${\text{s}}_{2}^{2}$ are the variance of context 1 and context 2’s band power in one session. The Fisher score is calculated independently for each channel, and its topology was obtained using FieldTrip (Oostenveld et al., 2010) toolbox; data in between-electrodes space are interpolated in a linear fashion.

When evaluating the statistical difference between the two groups, we noticed some outliers in subjects’ neurophysiological metrics (SMR predictor and control signal contrast); therefore, we identified and excluded these outliers from analysis with the same method mentioned in section “Performance Metric.” We did not find outliers when analyzing SMR predictor; we found additional two meditators and one control outlier when analyzing the resting-state EEG stability; we found an additional one meditator and one control outlier when analyzing the LR control signal; we found an additional one control outlier when analyzing the UD control signal. The results obtained in Figure 5B are obtained after excluding these additional outliers.

### Statistical Analysis

We performed linear mixed-effects models per type of performance and neurophysiological measures to investigate the session, group, and interaction effect. lme4 package (1.1-25) in R (4.0.3) was used to generate the linear mixed-effects models, and *p*-values were computed using lmerTest package (3.1-3), using Satterthwaite approximation for degrees of freedom (Kuznetsova et al., 2017). Each BCI performance and neurophysiological measure were modeled over time with a fixed effect of session (six levels) and group (two levels, meditator and control). Random effects include within-subject factors of the session. Models were initially fit with the interaction of group and time, and then, fixed effects were reduced stepwise by excluding non-significant interaction terms/predictors and compared using ANOVA ratio tests until this smaller model explained the data significantly worse than the larger model (significant Chi-squared test) (Kuznetsova et al., 2017). Other statistical tests used in this work include rank-sum test, linear regression, and Chi-squared tests; the details of these tests will be explained wherever it appears in section “Results.”

## Results

### Survey Results

**FIGURE 2:**
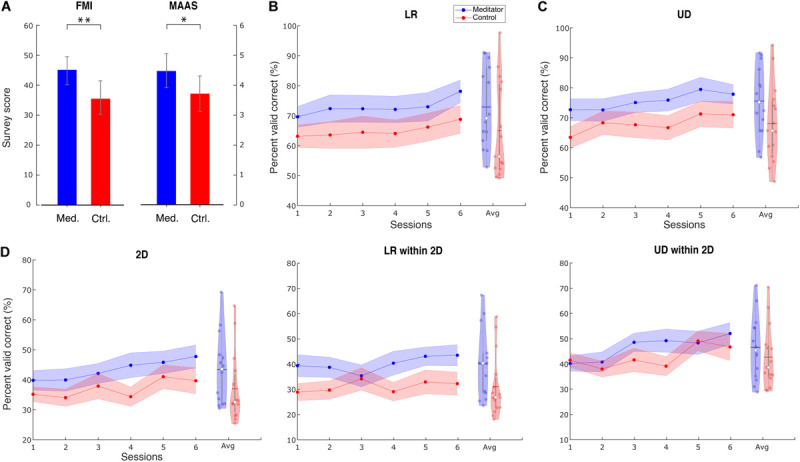
Survey results and group averaged performance and learning. **(A)** Survey results of FMI and MAAS show that meditators have a higher level of mindfulness than controls. Data are shown as mean ± *SD*. Med. is meditator, Ctrl. is control. **(B)** Line plot describes the group LR averaged PVC ± SEM for mediators and controls. Violin plot describes session-averaged performance distribution, with blue horizontal line indicating mean and white dot indicating median. **(C)** For UD task, **(D)** for 2D task, LR within the 2D task, and 2D within the 2D task. ^∗^ Indicates group difference with *p* < 0.05, and ^∗∗^ indicates *p* < 0.01, same for subsequent plots.

In both surveys, we found meditators had higher scores than control subjects. Concretely, the FMI score for meditators is 44.5 ± 4.5, whereas, for control subjects, it is 36.6 ± 6.7. The difference is significant (Wilcoxon rank-sum test, *Z* = 3.15, *p* < 0.01). The MAAS score for meditators is 4.42 ± 0.81, whereas, for control subjects, it is 3.73 ± 0.67. The difference is significant (Wilcoxon rank-sum test, *Z* = 2.53, *p* < 0.05). Bar plots for the two groups’ scores are shown in Figure 2A. The same observation also holds when including outlier subjects. These results serve as additional support, apart from the self-reported meditation experiences, that the meditators had higher levels of mindfulness than the control group. In addition to the group difference, we also calculated the correlation between these survey results and performance. We used baseline PVC as performance because this session is when the surveys were filled out. The correlation between survey results and UD PVC turned out to be significant. Specifically, for FMI, *r*(27) = 0.42, *p* < 0.05, and for MAAS, *r*(27) = 0.41, *p* < 0.05.

### Group Averaged Performance

We found that meditators achieved numerically better performance (PVC) compared with control subjects, and this difference was consistent throughout the six sessions. The group averaged performance in the baseline, and the final session is shown in Supplementary Table 3, and the averaged performance for all sessions is shown in Figure 2.

We used a linear mixed-effects model (see section “Materials and Methods”) to investigate the statistical difference between the two groups in terms of group, session, and group–session interaction effect. The session effect indicates if BCI learning occurs for a specific task. We found a significant learning effect in all three tasks [LR: *t*(144) = 2.98, *p* < 0.01; UD: *t*(28) = 2.22, *p* < 0.05; 2D: *t*(28) = 2.84, *p* < 0.01]. The difference in dimensionality is due to the difference in the final model when performing the stepwise reduction (see section “Materials and Methods”). However, the group effect did not show significance [LR: *F*(1, 27) = 2.01, *p* = 0.16; UD: *F*(1, 27) = 2.71, *p* = 0.11; 2D: *F*(1, 27) = 1.79, *p* = 0.19], indicating that there is only a numerical superiority of meditators’ BCI performance. We also did not find significance in the learning speed difference between the two groups, indicated by the interaction effect [LR: *F*(1, 143) = 0.1, *p* = 0.75; UD: *F*(1, 27) = 0.001, *p* = 0.97; 2D: *F*(1, 27) = 0.32, *p* = 0.57].

Given that the 2D task is the combination of LR and UD, we next separated the LR and UD tasks within the 2D. Interestingly, we found that within the 2D task, meditators had a numerically higher baseline of LR, but for the UD, these two groups were at the same level. Further, the learning curve showed that meditators had numerically better learning compared with controls in the UD within 2D. Statistical analysis using linear mixed-effects model shows that learning effect of UD within the 2D is significant, whereas LR within the 2D is not [LR within 2D: *F*(1, 28) = 2.58, *p* = 0.11; UD within 2D: *t*(28) = 2.92, *p* < 0.01]. We did not find the group effect to be significant [LR within 2D: *F*(1, 27) = 3.11, *p* = 0.08; UD within 2D: *F*(1, 27) = 0.45, *p* = 0.50], as well as the interaction effect [LR within 2D: *F*(1, 27) = 0.21, *p* = 0.64; UD within 2D: *F*(1, 27) = 0.28, *p* = 0.60].

### Competency Curve

Although group averaged PVC is a good indicator of performance, there are several drawbacks. First, it only provides information on the overall trend of performance during BCI learning; we still do not know how many subjects remain BCI inefficient. Second, it does not provide information regarding within-session learning.

**FIGURE 3:**
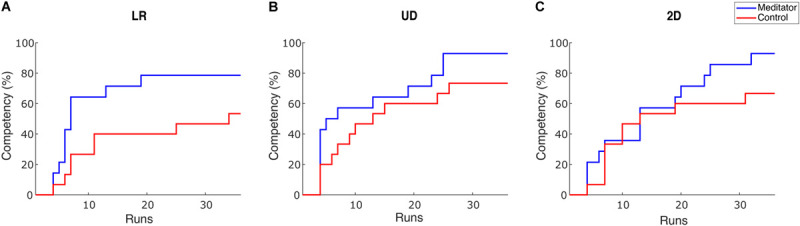
Competency curves for **(A)** LR, **(B)** UD and **(C)** 2D tasks. Competency describes the percentage of subjects passing the performance threshold (70% for 1D and 40% for 2D). Each session has 6 runs for one task, accounting for 36 runs in total throughout the 6 training sessions.

To intuitively show how learning occurs in the two groups, we plotted competency, the percentage of subjects whose PVC passed the BCI inefficiency threshold as sessions go on. We set the threshold as 70% for 1D control and 40% for 2D control (Combrisson and Jerbi, 2015), but we obtain similar results under varied thresholds. To cope with potential fluctuation of performance, a subject passes the threshold if she/he meets one of the following criteria: achieving an averaged PVC > threshold in three consecutive runs or achieving an averaged PVC > threshold in one single session (Cassady et al., 2014). The result is shown in Figure 3.

There are two observations from this plot. First, after six sessions of learning, the percentage of subjects passing the BCI inefficiency threshold appears to be higher in meditators. The percentage of non-BCI inefficient subjects is 78.5% (53.3%), 92.8% (73.3%), and 92.8% (66.7%) for meditators (controls), in LR, UD, and 2D tasks, respectively. Therefore, in all three tasks, meditators indeed had numerically less BCI inefficient subjects after six sessions or 36 runs of learning, but Chi-squared tests did not reveal a significant difference for the competency between two groups [X^2^(1, *N* = 29) = 2.04, 1.93, 3.02, *p* = 0.15, 0.16 and 0.08 for LR, UD, and 2D]. Second, regarding the speed of learning, the LR and the UD plot showed a steeper decline during the initial six runs, i.e., the baseline session. This means that the learning speed of meditators appears to be faster than the control subjects. Besides, although previous studies showed that BCI learning occurs on a session-by-session basis (Meng et al., 2016), our results showed that learning could also occur within a 2 h session. We also noticed that compared with 1D tasks (LR and UD), both groups in the 2D task showed a similar learning curve in the first 20 runs, i.e., in the first three sessions. After that, meditators showed a numerically better learning speed compared with control subjects. This observation is consistent with the previous group average performance in the sense that in UD within the 2D task, meditators had numerically larger improvement starting from the third session. In addition, it also shows that 2D control is indeed more difficult than 1D control, requiring more training time.

### Group Averaged Topology During Task

**FIGURE 4:**
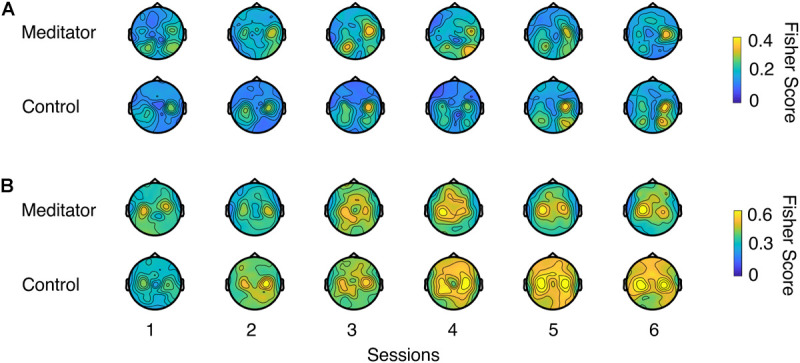
Fisher score topology for meditators and controls during **(A)** LR and **(B)** UD task as session goes on. Fisher score describes the mu rhythm contrast between two opposite contexts in a task (e.g., left trials and right trials in LR task, or up trials and down trials in UD task).

Figure 4 shows the LR and UD task Fisher score topology (Perdikis et al., 2018) for meditators and controls. From the plot, a gradual increase of motor cortex area high alpha power could be seen in both groups, indicating that both groups were able to increase the contrast of two opposite conditions through voluntary motor imagery as learning progresses. However, this plot did not provide quantitative information regarding whether meditators had a higher baseline of C3 and C4 high alpha power or exhibited better learning. We also did not find electrode clusters with a significant difference between the two groups with cluster-based permutation tests (Stieger et al., 2020). To further investigate the effect of meditation experience on these quantities, we looked into the SMR predictor during the intertrial resting state, mu power variability at rest, and control signal contrast during task execution.

### Neurophysiological Predictor

**FIGURE 5:**
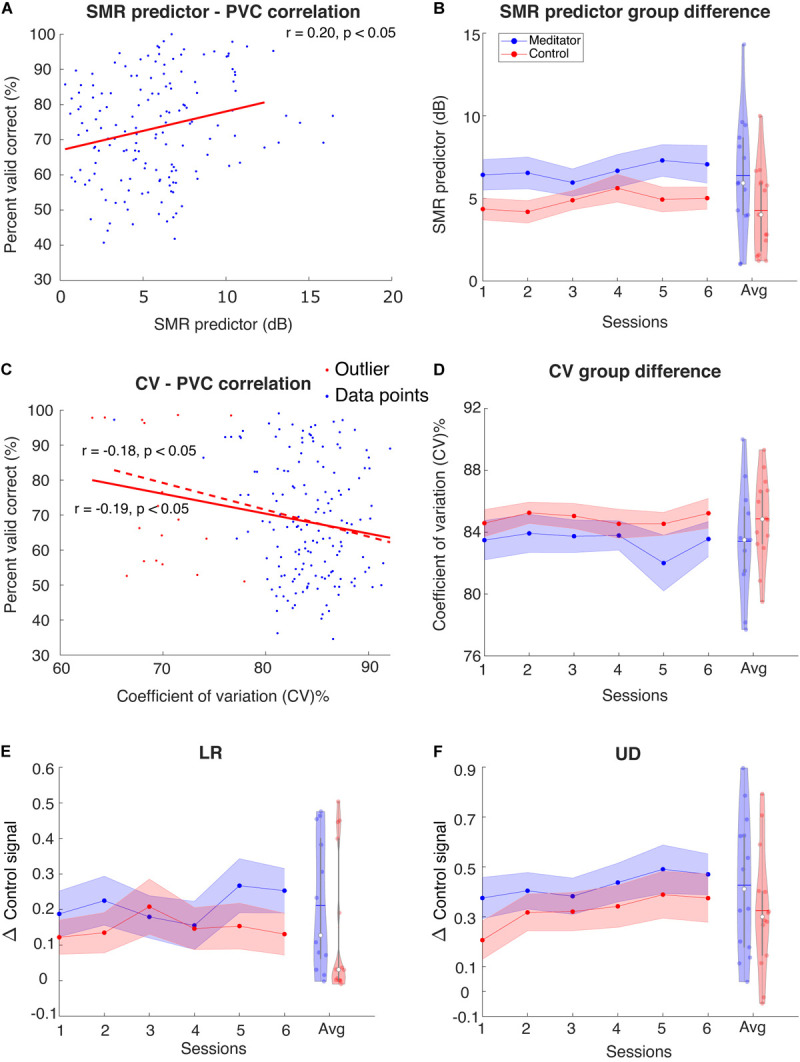
**(A)** Regression between SMR predictor and PVC for UD task. Red line is regression line. For LR the correlation is not significant (plot not shown). **(B)** Group averaged SMR predictor for the meditator group and control group. Violin plot shows the session-averaged result. **(C)** Regression between resting-state stability and PVC for LR task. For UD task the correlation is only significant before excluding outliers (plot not shown) **(D)** Group averaged stability for the meditator group and control group. Violin plot shows the session-averaged result. **(E,F)** Group averaged Δ control signal for **(E)** LR and **(F)** UD. Violin plot shows the session-averaged result.

Blankertz et al. (2010) found that in the resting state power spectral density plots of C3 and C4 electrodes, the difference between mu rhythm peak and noise level baseline is a significant predictor of the BCI performance. Here, we tried to investigate the difference in SMR predictor between meditators and controls. As shown in Figure 5A, we first fit a linear regression model between the SMR predictor and PVC. We found that in the LR task, the correlation coefficient between SMR predictor and PVC is *r*(153) = 0.13, *p* = 0.11, and in the UD task, *r*(153) = 0.20 with *p* < 0.05. Our correlation coefficient was smaller than that of Blankertz et al. (2010). The difference might be due to the task design, subject variability, or it could be due to the fact that Blankertz et al. (2010) recorded a 2 min resting state, whereas, here, we used multiple short pretrial segments. We next asked if the session, group, and interaction effects exist in the SMR predictor. We found that the session effect is significant [*t*(125) = 2.42, *p* < 0.05], but we did not find the group and interaction effects to be statistically different between meditators and controls [group effect: *F*(1, 27) = 3.29, *p* = 0.08; interaction: *F*(1, 124) = 0.001, *p* = 0.96]. However, as shown in Figure 5B, a numerical difference between the meditator group and the control could be observed.

### Variability of Resting Electroencephalogram Mu Rhythm

Another perspective of investigating the resting state difference between meditators and controls is the stability of the EEG pattern, i.e., the mu rhythm in the SMR BCI setting. Sannelli et al. (2008) pointed out that BCI inefficient subjects usually have higher intrinsic noise. Specifically, this means that the presentation of noise in EEG band power overshadows the useful information. In our study, a relevant measurement of noise could be the variability or stability of EEG mu rhythm, and we hypothesize that this is related to the performance. Here, we used the coefficient of variation (CV) (Brown, 1998) to measure variability: the ratio of intertrial resting-state EEG mu power standard deviation and its mean. The lower the value, the more stable the EEG pattern is. We indeed found that the stability is negatively correlated with the performance [LR: *r*(172) = −0.19, *p* < 0.05, UD: *r*(172) = −0.18, *p* < 0.05], when excluded potential outlier points, the relationship between LR PVC and stability is still significant [LR: *r*(154) = −0.17, *p* < 0.05], whereas for UD, it is not [UD: *r*(154) = −0.13, *p* = 0.11], indicating that the correlation is weak, especially for UD task. This CV did not show session, group, or interaction effect [session effect: *F*(1, 129) = 0.34, *p* = 0.55; group effect: *F*(1, 24) = 1.36, *p* = 0.25; interaction: *F*(1, 128) = 0.63, *p* = 0.42]. These results are shown in Figures 5C,D.

### Control Signal Baseline and Learning

Given the behavior difference described in the previous section, the next question to ask is whether meditators exhibit better overall performance and learning of Δcontrol signal. Like the online scenario (see section “Materials and Methods”), we computed the control signal as the difference of z-scored C4 power and C3 power (for LR) or summation of z-scored C4 power and C3 power. The Δcontrol signal is the difference of control signal between two opposite trial types (LR and UD). Figures 5E,F show the group averaged Δcontrol signal as sessions go on. We noticed a session effect in the UD Δcontrol signal [*t*(27) = 2.05, *p* < 0.05] but not the LR Δcontrol signal [*F*(1,134) = 0.96, *p* = 0.32]. However, we did not notice significance in group [LR: *F*(1,25) = 0.68, *p* = 0.41; UD: *F*(1, 26) = 1.26, *p* = 0.27] or interaction effect [LR: *F*(1,133) = 0.71, *p* = 0.39; UD: *F*(1, 26) = 0.09, *p* = 0.75].

## Discussion

Reducing the training time and BCI inefficiency is critical for the application of SMR-based BCI. Although prior studies have tried to solve this problem from the “brain” side of BCI by investigating the effect of meditation experience on SMR BCI learning, the relationship between these two is still not comprehensive. First, due to the large variability in the type and duration of meditation, more studies are needed to confirm the existence of such an effect. Second, it is still unclear whether and to what extent meditators are better able to do more complex tasks than 1D control. Third, a more thorough investigation of the neurophysiological difference between these two groups is needed.

Our results provide insights into the effect of long-term meditation experiences on SMR-based BCI. Concretely, we found that level of mindfulness is significantly correlated with the SMR BCI performance in the UD task, and experienced meditators had numerically higher overall BCI performance compared with meditation naïve subjects. We also found that there were numerically fewer BCI inefficient subjects remaining after six sessions of learning. As for task complexity, we extended the control paradigm to a more complex 2D cursor control task. We found a similar trend when separating the LR and UD tasks within the 2D control, that meditators had numerically higher LR performance within the 2D task than controls. We also found that although meditators and controls started at approximately the same level of UD performance within the 2D task, numerically, meditators exhibited better learning and resulted in higher improvement than controls. Finally, neurophysiology analysis revealed a numerical difference between the SMR predictor, resting mu power stability, and UD control signal. Nevertheless, the statistical significance mainly lies in the learning of the task, i.e., subjects statistically improved their BCI performance after learning; we did not find that meditators statistically outperformed control subjects in terms of averaged performance and learning speed. As for the task difficulty and learning, we found that although the 1D version of LR and UD tasks both have a significant learning effect, in 2D, only the UD part showed significant learning. In terms of the Δcontrol signal, we also found a significant learning effect of UD rather than LR. This observation is in agreement with Stieger et al. (2020) and suggests that the neurophysiological processes involved in learning the UD task (motor imagery vs. rest) could be easier to learn than the LR task (left motor imagery vs. right motor imagery).

It should be noted that our experimental task is consistent with prior work (Cassady et al., 2014) in terms of the platform (BCI 2000) and 1D BCI task design. However, there are several points that this prior work did not address: (1) Most of their claims were focused on the behavioral difference, including PVC and competency; the analysis on the neurophysiological difference between the two groups was limited. We added the electrophysiological topology, SMR predictor, mu stability, and control signal analysis to this framework, which could better explain the neurophysiological difference between the two groups. (2) Cassady and colleagues’ work did not implement 2D control tasks, which represents a more challenging task. Although by design, the 2D task is the combination of LR and UD control, in real-time BCI control, it is more challenging because subjects need to carefully maintain the cursor position while moving the cursor in the prompted direction. As meditators are better able to control their attention, they might perform even better than controls compared with 1D tasks. Currently, we are not aware of prior literature explicitly investigating the 2D cursor control of long-term meditators and controls. Therefore, it is of interest to see if meditators would also be better at the 2D task, how much they outperform the controls, and if there is any difference between the LR and UD within the 2D compared with the 1D version of LR and UD tasks. (3) It is known that there exists a large variability of SMR BCI performance in the population. Apart from the literature that reports the positive influence of meditation on SMR BCI control, we are also aware of literature that only partially supports or does not support the hypothesis that people with meditation experience would demonstrate better SMR BCI control (Botrel and Kübler, 2019; Stieger et al., 2020). Therefore, to make a claim more rigorous, it is important to show the replicability of results.

Our study serves to confirm and extend this finding by conducting experiments at a different location and time with independent subjects and experimenters. Specifically, although we generally found that meditators on average outperformed control subjects by 5–10% PVC, we did not find a statistical difference between the two groups in terms of overall performance (as shown in the group effect) and learning speed (as shown in the interaction effect), and the percentage of BCI inefficient subjects in these two groups was not statistically different (although the *p*-value was small). The difficulty of getting statistical significance could be due to the following two reasons: (1) Meditation experience is not the determining factor for generating SMR, and the effect is weak. (2) The variability among SMR BCI performance is large, thus requiring a larger sample size. For example, in Stieger et al. (2020), they implemented twice as large a sample size as us, but this requires much larger efforts to acquire. Therefore, our work updates the community about “how much” meditators perform better than meditation naïve people at SMR BCI and could serve as a reference for researchers who would like to recruit experienced meditators to obtain a better SMR BCI control.

Nevertheless, it is still of interest to discuss the potential cause of this meditation effect, as it could provide insight into what factors do influence BCI performance: The long-term meditation effect on SMR BCI could be due to the plasticity introduced by meditation experience. For example, one of the main benefits of mindfulness meditation is enhanced attentional control (MacLean et al., 2010). In the SMR BCI task, subjects are instructed to focus on or pay attention to the motor intention, which could be regarded as a specific type of attention control. Therefore, the prolonged meditation practices might serve as additional “training time” and cause the meditator group to have enhanced BCI performance. Future work along this line should investigate if neurotypical people are also able to improve SMR BCI control, apart from UD tasks (Stieger et al., 2020), with more extended meditation training.

An alternative explanation would be the preexisting difference in brain structure, personality, etc., for people who choose to meditate for years (Tang et al., 2015). In other words, the subpopulation who choose to meditate for years may have attributes that contribute to a successful SMR BCI control. Nevertheless, the research focusing on SMR BCI control ability for people with different characteristics is still limited, and future work on investigating the impact of these multidimensional and interrelated personal attributes might reveal more details of SMR BCI control.

The presence of BCI proficient subjects is also of importance to study. Multiple studies have shown that there exists a certain portion of subjects who are able to control the SMR BCI with very high accuracy the first time they use this technology (Edelman et al., 2019; Stieger et al., 2020). In our study, we quantified these subjects by the outlier exclusion criteria described in section “Materials and Methods.” See Supplementary Table 2 for the details of these outliers’ performance. We found five subjects quantified as outliers; all were BCI proficient subjects. Interestingly, we found that there is only one meditator but four controls among them. This phenomenon is interesting because (1) it points out that meditation is not the determining factor of BCI proficiency, as a large portion of outliers are controls; (2) given that controls have numerically lower BCI performance and more BCI proficient subjects, larger variability might exist within the population with no meditation experience. Although more data are needed to validate this observation, it could be another perspective to investigate the effect of meditation on SMR BCI.

EEG resting mu rhythm variability and SMR BCI. In this study, we found that the resting EEG coefficient of variation (CV) is related to SMR BCI performance and could serve as an SMR BCI indicator, such as the SMR predictor (Blankertz et al., 2010). However, going back to the central question of finding a training paradigm to help prepare subjects for SMR BCI control, such an indicator is not optimal due to the lack of a clear training method, i.e., the training procedure of reducing resting EEG variance is not well established. We believe at least two potential research directions could be inspired by our work given the numerically more stable resting-state EEG signals of the meditator group: (1) Validation of the effect of resting-state EEG signal on SMR BCI performance by an independent investigator and BCI system; (2) A further investigation of the relationship between meditation training and resting-state EEG stability through a longitudinal perspective, i.e., if people could gain more stable resting-state EEG signal through meditation training. Research along this line could answer both “what causes the SMR BCI performance variation” and “how to improve SMR BCI,” which we believe is of high practical value.

Potential influence of presentation of the three tasks. In our study, the order of the three tasks is fixed for all subjects, i.e., LR followed by UD, followed by 2D. The fact that they are not randomized could influence the performance because subjects are usually more concentrated on the early phase of the experiment. Nevertheless, we believe the current study design is still of benefit to the question we are trying to address: if there exists a difference in learning within a task between meditators and controls. It would be fairer to compare the performance of a task given a similar level of tiredness. On the other hand, the randomized task design could be used to more rigorously investigate if the learning between different tasks is different, but it should also be noted that a larger sample size would be needed because of the randomized design.

Another concern regarding studying these two distinct groups is the effect of age and sex on our results. Although we tried our best to find age-matched controls for the meditators, the meditators were on average 38.5 years old, and the controls were on average 24.8 years with a 13.7 years difference. One might argue that if meditators in our sample were more senior, this might affect our conclusion. However, we did not find evidence of significant correlations between age and performance, age and Δcontrol signal, or age and SMR predictor (see Supplementary Table 4). These results suggest that the influence of age on our BCI system is not significant. As for sex, we have six females and eight males in the meditator group and 11 females and four males in the control group. Randolph (2012) found that females could be better at BCI tasks, but in our BCI setting, we did not find a significant difference in LR, UD, and 2D performance or SMR predictor between male and female subjects (see Supplementary Table 4). Nevertheless, this insignificance could also be due to the insufficient sample size, and future work along this line should either try to recruit a larger number of samples to validate the effect of age and sex or try to recruit subjects with a more balanced age and sex.

## Conclusion

In this study, we have examined the behavior and neurophysiological differences between experienced meditators and control subjects. We found evidence supporting that long-term meditation experiences could influence SMR BCI in terms of averaged performance, SMR predictor, resting-state mu stability, and control signal during task execution. This finding has implications on enhancing the “brain” side of SMR BCI and may help overcome the limitations of SMR BCI technology, such as long training time and BCI inefficiency.

## Data Availability Statement

The data presented here are available upon reasonable request from the corresponding author.

## Ethics Statement

The studies involving human participants were reviewed and approved by the Institutional Review Board (IRB) of Carnegie Mellon University. The participants provided their written informed consent to participate in this study.

## Author Contributions

XJ was involved in experiment conduction, data analysis, and manuscript writeup. EL was involved in experiment conduction and manuscript review. JS was involved in study design, experiment conduction, and manuscript review. CG was involved in study design, subject recruitment, and manuscript review. BH was involved in the conception, study design, supervision, and manuscript review. All authors contributed to the article and approved the submitted version.

## Conflict of Interest

The authors declare that the research was conducted in the absence of any commercial or financial relationships that could be construed as a potential conflict of interest.

## Supplementary Material

Supplementary Figure 1 Comparison of two methods to compute the Δcontrol signal during task execution. The traditional method to quantify how EEG band power changes during task execution is event-related desynchronization. Concretely, the control signal under the ERD definition would be band power normalized by the resting state alpha activity. Here we argue that the control signal using the z-score method would be a better metric by showing that it explains more performance variability. **(A)** in LR, the correlation coefficient for regression between Δcontrol signal and PVC was 0.70 in the ERD method and 0.8 in the z-scored method, *p* < 0.05, **(B)** for UD it was 0.67 and 0.82, *p* < 0.05.

Supplementary Figure 2 Violin plot for performance and SMR. The violin plot provides more detailed information regarding the mean, median and distribution of the data. **(A)** for LR PVC, **(B)** for UD PVC, **(C)** for 2D PVC, **(D)** for SMR predictor, **(E,F)** for LR and UD Δ control signal. The blue/red dots represent everyone’s performance, the white dot indicates the median, the blue/red horizontal lines represent the mean, and the violin-like envelop represents the distribution density.
