# Supplementary figures and images for: Effects of Long-Term Meditation Practices on Sensorimotor Rhythm-Based Brain-Computer Interface Learning
Source: Front Neurosci. 2021 Jan 21;14:584971. doi: 10.3389/fnins.2020.584971 (PMC7858648; doi:10.3389/fnins.2020.584971)

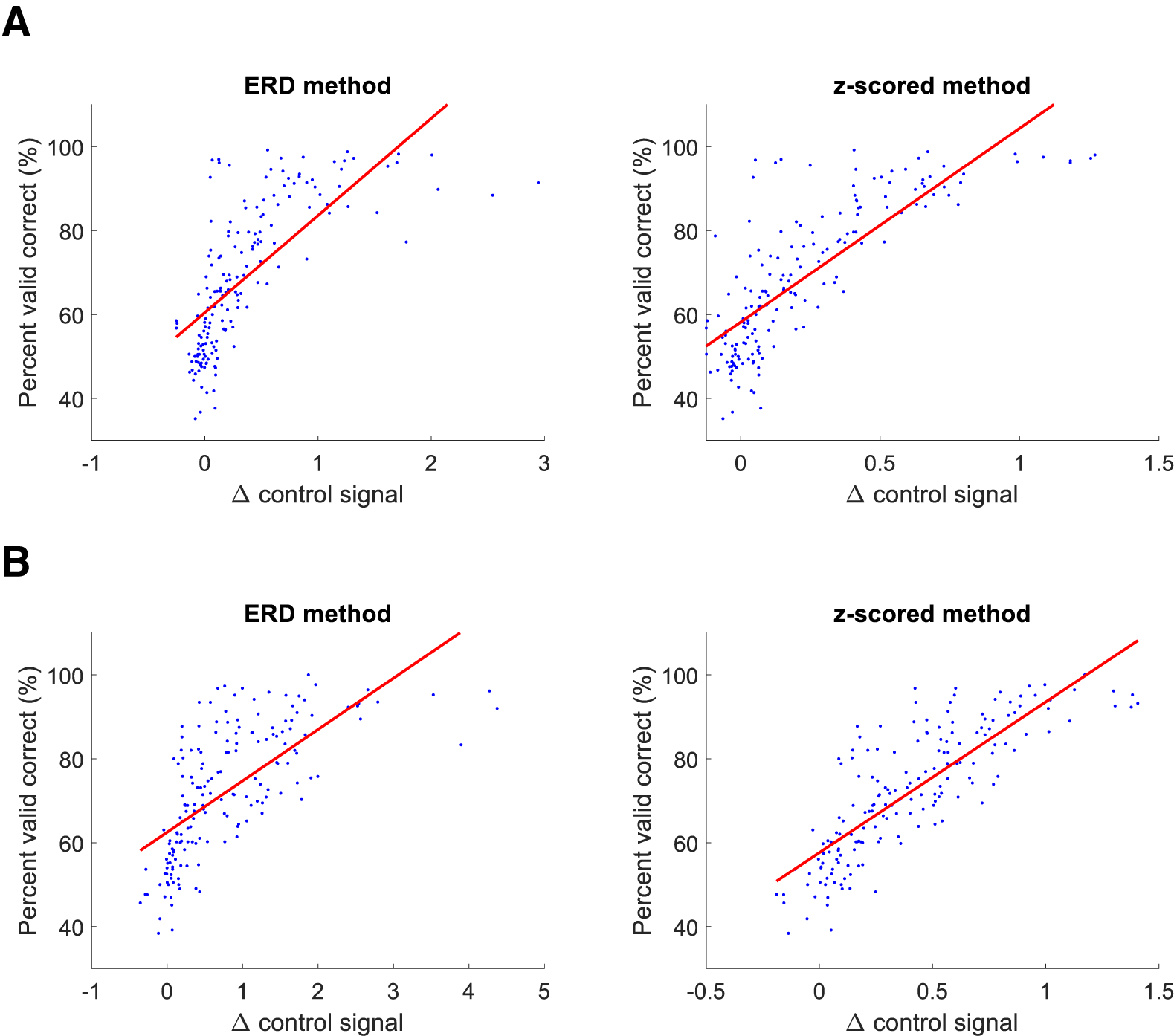

Supplement: Supplementary Figure 1 — Comparison of two methods to compute the Δcontrol signal during task execution. The traditional method to quantify how EEG band power changes during task execution is event-related desynchronization. Concretely, the control signal under the ERD definition would be band power normalized by the resting state alpha activity. Here we argue that the control signal using the z-score method would be a better metric by showing that it explains more performance variability. (A) in LR, the correlation coefficient for regression between Δcontrol signal and PVC was 0.70 in the ERD method and 0.8 in the z-scored method, p < 0.05, (B) for UD it was 0.67 and 0.82, p < 0.05. [file Image_1.tif]

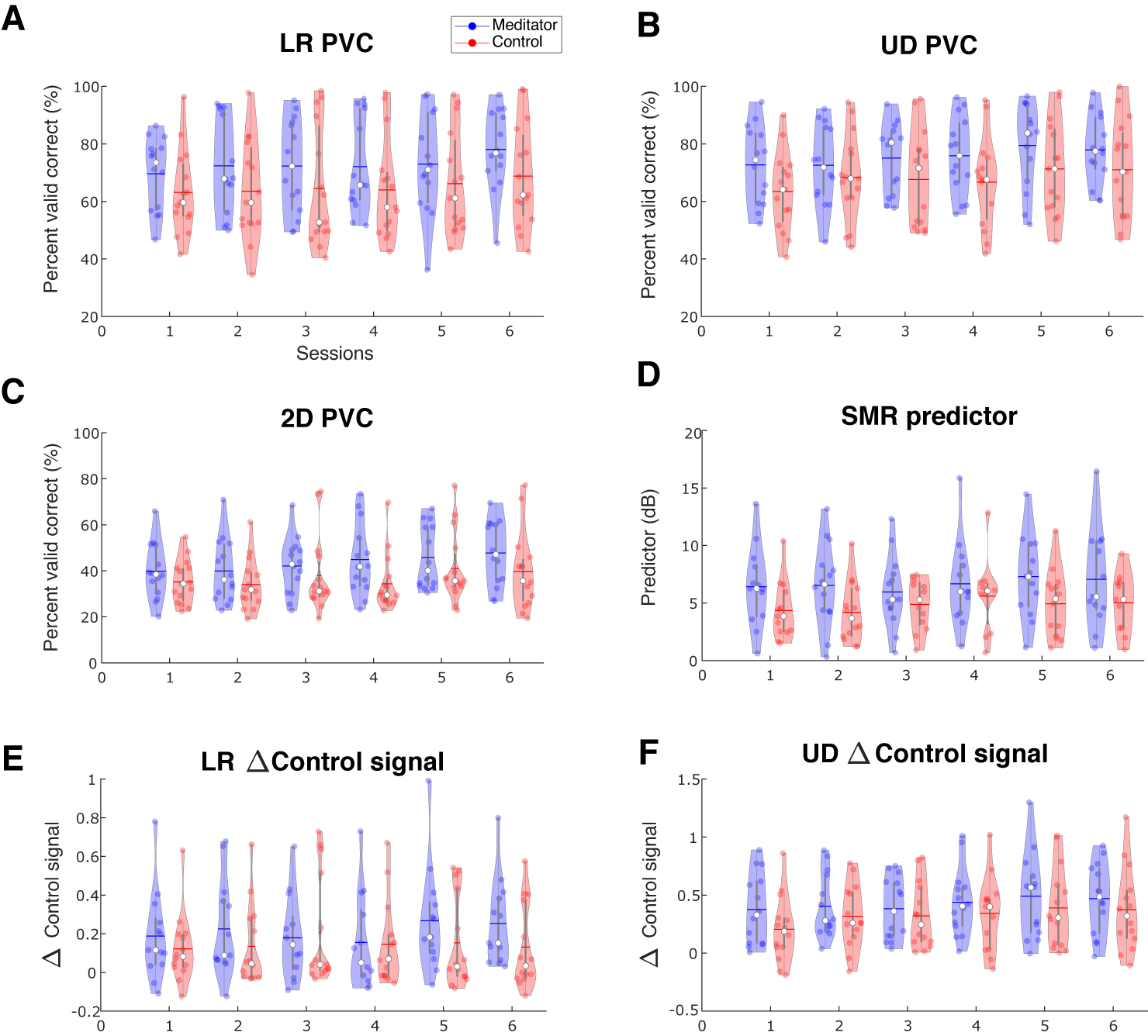

Supplement: Supplementary Figure 2 — Violin plot for performance and SMR. The violin plot provides more detailed information regarding the mean, median and distribution of the data. (A) for LR PVC, (B) for UD PVC, (C) for 2D PVC, (D) for SMR predictor, (E,F) for LR and UD Δ control signal. The blue/red dots represent everyone’s performance, the white dot indicates the median, the blue/red horizontal lines represent the mean, and the violin-like envelop represents the distribution density. [file Image_2.tif]
