# Supplementary material for: Effects of Long-Term Meditation Practices on Sensorimotor Rhythm-Based Brain-Computer Interface Learning
Source: Front Neurosci. 2021 Jan 21;14:584971. doi: 10.3389/fnins.2020.584971 (PMC7858648; doi:10.3389/fnins.2020.584971)
Supplement: Supplementary file 3 [file Table_1.docx]

Table S1. Subject demographic information and survey results. FMI indicated Freiburg Mindfulness Inventory; MAAS indicated Day-to-day experience survey. Recruited subjects are all the subjects recruited in this study, and analyzed subjects are subjects that subjects included in the quantitative analysis after performing the outlier exclusion procedure (see Methods). The age, FMI result, MAAS result in this table is obtained after excluding outliers.

|  | Meditator (16 recruited, 14 analyzed) | Control (19 recruited, 15 analyzed) |
| --- | --- | --- |
| Demographics (Number) | Recruited: White(12), Black(1), Asian(2), White/Indian(1)  Analyzed: White(10), Black(1), Asian(2), White/Indian(1) | Recruited: White(13), Black(2), Asian(3), Hispanic(1)  Analyzed: White(9), Black(2), Asian(3), Hispanic(1) |
| Age (± std)  (analyzed subjects only) | 38.5 ± 15.7 | 25.6 ± 9.4 |
| Gender | Recruited: F = 6, M = 10  Analyzed: F = 6, M = 8 | Recruited: F = 14, M = 5  Analyzed: F = 11, M = 4 |
| FMI result (± std)  (analyzed subjects only) | 44.5 ± 4.5 | 36.6 ± 6.7 |
| MAAS result (± std)  (analyzed subjects only) | 4.42 ± 0.81 | 3.73 ± 0.67 |
