# Supplementary material for: Effects of Long-Term Meditation Practices on Sensorimotor Rhythm-Based Brain-Computer Interface Learning
Source: Front Neurosci. 2021 Jan 21;14:584971. doi: 10.3389/fnins.2020.584971 (PMC7858648; doi:10.3389/fnins.2020.584971)
Supplement: Supplementary file 4 [file Table_2.docx]

Table S2. Median +/- median absolute deviation of all subjects recruited and outlier subject’s performance, related to the outlier exclusion method. The first three rows are all recruited subjects’ data (including outliers) and are shown as median ± median absolute deviation. The last row only shows the performance of each individual outlier. The first outlier is a meditator while the rest are controls.

| Identity \ PVC (%) | LR | UD | 2D |
| --- | --- | --- | --- |
| All sample | 69.1 ± 15.7 | 73.4 ± 12.8 | 40.5 ± 8.6 |
| Meditator | 70.4 ± 12.1 | 78.0 ± 11.7 | 44.6 ± 11.6 |
| Control | 66.4 ± 15.9 | 72.9 ± 15.7 | 33.3 ± 6.4 |
| Outliers only | 95.8, 96.2, 97.9, 96.0, 100.0 | 95.3, 90.8, 95.9, 91.8, 95.8 | 79.4, 77.2, 73.2, 74.8, 85.1 |
