# Supplementary material for: Effects of Long-Term Meditation Practices on Sensorimotor Rhythm-Based Brain-Computer Interface Learning
Source: Front Neurosci. 2021 Jan 21;14:584971. doi: 10.3389/fnins.2020.584971 (PMC7858648; doi:10.3389/fnins.2020.584971)
Supplement: Supplementary file 5 [file Table_3.docx]

Table S3. Group averaged performance (PVC) from baseline and final session

| Meditator | Baseline | 69.6 | 72.7 | 39.8 |
| --- | --- | --- | --- | --- |
|  | Final | 78.1 | 77.8 | 47.7 |
| Control | Baseline | 63.1 | 63.4 | 35.1 |
|  | Final | 68.8 | 71.4 | 39.7 |
