# Supplementary material for: Effects of Long-Term Meditation Practices on Sensorimotor Rhythm-Based Brain-Computer Interface Learning
Source: Front Neurosci. 2021 Jan 21;14:584971. doi: 10.3389/fnins.2020.584971 (PMC7858648; doi:10.3389/fnins.2020.584971)
Supplement: Supplementary file 6 [file Table_4.docx]

Table S4. Statistical report for the influence of age and gender on performance and SMR

|  | Statistical test report |
| --- | --- |
| Age and PVC | Pearson correlation, r(27) = 0.03, 0.13, -0.02 with p = 0.86, 0.47, 0.89 for LR, UD and 2D task |
| Age and ∆control signal | Pearson correlation, r(27) = -0.11 and p = 0.57. |
| Age and SMR predictor | Pearson correlation, r(27) = -0.21, -0.22 and p = 0.25, 0.23 for LR and UD ∆control signal |
| Gender and PVC | Wilcoxon Rank-Sum Test, Z = -0.37, 0.15, 0.51, p = 0.71, 0.87, 0.61 for LR, UD and 2D PVC |
| Gender and ∆control signal | Wilcoxon Rank-Sum Test, Z = 1.12, p = 0.25 |
| Gender and SMR predictor | Wilcoxon Rank-Sum Test, Z = -0.06, 0.95, p = 0.94, 0.34 for LR, UD ∆control signal |
